# A case of stent-assisted balloon-induced intimal disruption and relamination of distal remaining aortic dissection after the ascending aorta and aortic arch replacement for acute aortic dissection

**DOI:** 10.1186/s44215-023-00071-0

**Published:** 2023-09-14

**Authors:** Seito Shimizu, Yujiro Kawai, Yuki Horinouchi, Ayaka Yu, Masanori Hayashi, Kanako Kobayashi, Takahito Itoh, Naoki Fujimura, Hirohisa Harada, Satoshi Ohtsubo

**Affiliations:** 1 Department of Cardiovascular Surgery, Tokyo Saiseikai Central Hospital, Tokyo, Japan; 2 Department of Vascular Surgery, Tokyo Saiseikai Central Hospital, 1-4-17 Mita, Tokyo, 108-0073 Japan

**Keywords:** Acute aortic dissection, Thoracic endovascular aortic repair (TEVAR), Stent-assisted balloon-induced intimal disruption and relamination of aortic dissection (STABILISE)

## Abstract

**Background:**

The early survival rate of patients with acute type A aortic dissection (TAAD) has improved remarkably over the past two decades. However, a false lumen may remain after proximal aortic repair and is a potential risk factor for aortic diameter enlargement, aortic rupture, and death in the chronic phase. In the stent-assisted balloon-induced intimal disruption and relamination of aortic dissection (STABILISE) technique, a stent graft is implanted at the proximal part of the aortic dissection, followed by bare-metal aortic stent placement over the distal aortic dissection. Next, an aortic balloon catheter is dilated inside the stent graft and the bare stent to disrupt the intima and collapse the false lumen, immediately restoring uniluminal thoracoabdominal aortic flow. This report describes our experience with the STABILISE technique for a residual aortic dissection after an ascending aorta, and partial aortic arch replacement for acute TAAD resulted in complications.

**Case presentation:**

A 60-year-old man presented to our hospital with chest pain, paresthesia, and left lower-limb paralysis. He was diagnosed with acute type A aortic dissection (TAAD) and malperfusion of the left lower limb by computed tomography (CT), and an emergent ascending aorta and partial aortic arch replacement were performed. Immediately after surgery, the left femoral arterial pulse improved, and left lower limb revascularization was deferred. The postoperative course was good; however, back pain and intermittent claudication recurred on the 9th day after surgery. A CT examination revealed persistent antegrade false lumen flow in the descending thoracic aorta through the left subclavian artery dissection and retrograde false lumen flow from the thoracoabdominal segment, resulting in aortic diameter enlargement and narrowing of the true lumen. The symptoms did not improve, and the STABILISE technique was performed on the 12th day after the initial aortic surgery. The patient’s subsequent course was good; the back pain and intermittent claudication resolved. The patient was discharged on the 8th day after the STABILISE technique.

**Conclusions:**

The STABILISE technique can obliterate the false lumen and resolve symptoms associated with a residual false lumen after proximal aortic repair for acute TAAD.

## Background

The early survival rate for acute type A aortic dissection (TAAD) has improved remarkably over the past two decades. However, a false lumen may remain in the descending and abdominal aortas after proximal aortic repair. A residual false lumen can cause problems, such as rapid dilatation of the aorta, narrowing of the true lumen, and malperfusion in the acute phase. For instance, Park et al. reported an abdominal aortic false lumen patency of up to 89% after acute DeBakey type 1 aortic dissection repair [1]. Furthermore, persistent patency of a false lumen of the thoracoabdominal aorta has been reported as a risk factor for aortic diameter enlargement, aortic rupture, and death in the chronic phase [2]. Therefore, obliterating the distal aortic false lumen after proximal aortic repair may improve outcomes.

Hofferberth et al. first reported the stent-assisted balloon-induced intimal disruption and relamination of aortic dissection (STABILISE) technique, which can obliterate the false lumen, in 2014 [3]. In the STABILISE technique, a stent graft is implanted at the proximal part of the aortic dissection to close the entry tear, followed by bare-metal aortic stent placement over the distal aortic dissection. Next, an aortic balloon catheter is dilated inside the stent graft and the bare stent to forcefully disrupt the intima and collapse the false lumen, immediately restoring uniluminal thoracoabdominal aortic flow.

This report describes our experience implementing the STABILISE technique for residual aortic dissection after complications from an ascending aorta and partial aortic arch replacement for acute TAAD.

## Case presentation

A 60-year-old man presented at our hospital with sudden chest pain. The patient was a current smoker (five cigarettes per day) and had hypertension, dyslipidemia, and a history of vertebrobasilar artery dissection. A physical examination on arrival revealed coldness and pallor of the left lower limb. The left dorsal foot artery was not palpable, and the patient could not move their left lower limb. Computed tomography (CT) revealed TAAD extending to the bilateral external iliac arteries (EIA) with primary entry just above the Valsalve’s sinus. Cardiac tamponade or visceral artery malperfusion was not observed. The dissection involved the brachiocephalic artery (BCA), right common carotid artery (CCA), and right and left subclavian arteries (SCAs). The true lumen of the left EIA was severely collapsed because of the false lumen, and thus, a contrast exam of the common femoral artery (CFA) was delayed.

The patient underwent emergent ascending aorta and partial aortic arch replacement with BCA and left CCA reconstruction. The aorta was resected 1 cm above the sinotubular junction and then transected. A tear was observed in the lesser curvature of the aortic arch just below the left CCA, which was considered the primary entry. Then, the primary entry was resected, and the aortic arch was transected in zone 2. A 4-branched J-graft® (24 mm, Japan Lifeline, Tokyo, Japan) was selected, and the ascending aorta and partial aortic arch were replaced with the graft.

**Fig. 1:**
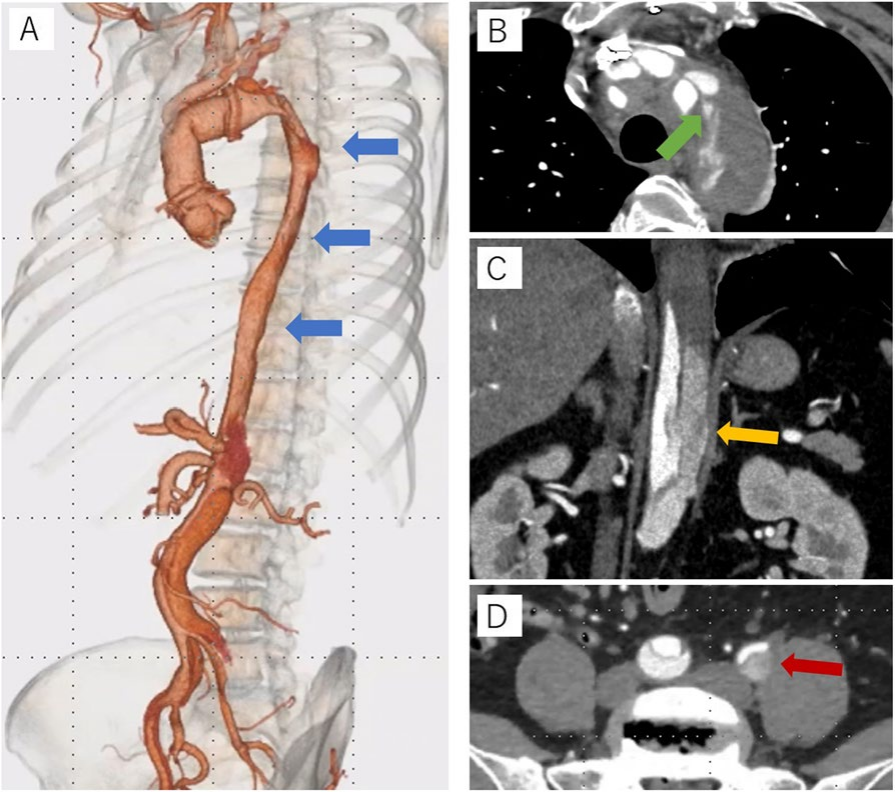
**A**–**D** CT images before STABILISE technique. Legend: **A** 3D image of the CT scan 9 days after the proximal aortic repair. True lumen of the descending thoracic aorta was narrowed due to compression by the false lumen (blue arrow). **B** Axial image at the level of the left SCA ostium. Residual dissection in the left SCA along with antegrade blood flow into the false lumen of the aortic arch was observed (green arrow). **C** Coronal image of the thoracoabdominal aorta. Retrograde blood flow into the false lumen of the thoracoabdominal aorta to descending aorta was observed (yellow arrow). **D** Axial image at the level of the EIA. The true lumen of the left EIA is narrowed due to compression by the false lumen (red arrow)

Immediately after surgery, the left femoral arterial pulse improved, and additional revascularization of the left lower extremity was deferred. There was no intermittent claudication after the surgery, and the postoperative course was good. However, on the 9th day after surgery, back pain and intermittent claudication of the left lower limb reemerged, so a follow-up CT scan was performed. The primary entry at the aortic arch was closed; however, a persistent antegrade false lumen flow in the descending thoracic aorta was present through the left SCA dissection, along with retrograde false lumen flow from the thoracoabdominal segment, resulting in enlargement of the aortic diameter to 42 mm at the distal arch and narrowing of the true lumen (Fig. 1 A–D). Furthermore, the left EIA had a significantly narrowed true lumen, and an ankle brachial pressure index (ABI) exam performed on the 10th day after surgery showed a decreased value of 0.53 (normal range: 0.90–1.40) on the left side. As for the visceral arteries, there was no dissection present, and only left renal artery was perfused from both true lumen with all other visceral arteries diverging from true lumen.

**Fig. 2:**
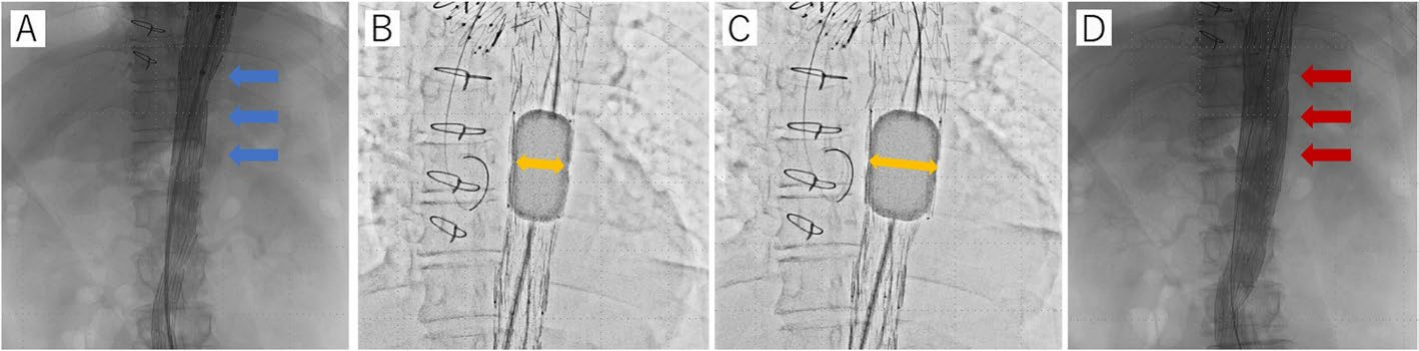
**A**–**D** Intraoperative imaging of STABILISE technique. Legend: **A** The pre-STABILISE-angiography showed narrowing of the true lumen and the stent graft (blue arrow). **B** Just before the dilatation of the balloon (yellow arrow). **C** Just after dilatation of the true lumen with the balloon disrupting the intima (yellow arrow). **D** The post-STABILISE angiography showed favorable dilatation of the true lumen (red arrow)

The patient underwent reoperation using the STABILISE technique on the 12th day after the initial surgery since the symptoms did not improve. The patient first underwent a debranching procedure from the left CCA to the left SCA bypass using 8-mm Propaten with a ring (WL Gore, Flagstaff, AZ, USA). Since the aortic diameter was 26 mm at the proximal landing zone (= J-graft) and 29–32 mm at the distal descending aorta, a Zenith TXD® 32–200 mm (Cook Medical, Bloomington, IN, USA) was selected and deployed just distal to the left CCA branch of the J-graft, closing the left SCA. An additional stent graft (Zenith Alpha® 32–80 mm, Cook Medical) was deployed with a 1.5 stent overlap from the previous stent graft. The first bare-metal aortic stent (Zenith Dissection® 36–123 mm, Cook Medical) was deployed from the second stent graft to just proximal to the celiac artery, and the second Zenith Dissection® 36–164 mm stent (Cook Medical) was deployed with a 25-mm overlap from the first Zenith dissection® stent to just above the aortic bifurcation. Pre-STABILISE angiography showed narrowing of the true lumen with all visceral arteries patent (Fig. 2A). The distal part of the stent graft was carefully dilated with a Coda Balloon (Cook Medical) and easily dilated to the intended diameter. The STABILISE technique seemed feasible from the initial dilation, and it was carefully performed over the entire length of the Zenith Dissection® (Fig. 2 B, C). Post-STABILISE angiography revealed favorable true lumen dilation and a completely obliterated false lumen (Fig. 2D). However, angiography also revealed occlusion of the left renal artery and bail-out stenting using the Express™ SD stent 6 mm × 18 mm (Boston Scientific, Marlborough, MA, USA) was performed. Next, aortic angiography was performed again, which confirmed the patency of all visceral arteries. Finally, angiography of the iliac arteries confirmed the residual left common iliac artery dissection and severe narrowing of the left EIA true lumen. To treat the EIA, an S.M.A.R.T.® stent 8 mm × 10 cm (Cordis Japan, Tokyo) was implanted from the ostium of the EIA; angiography confirmed good dilation of the EIA.

**Fig. 3:**
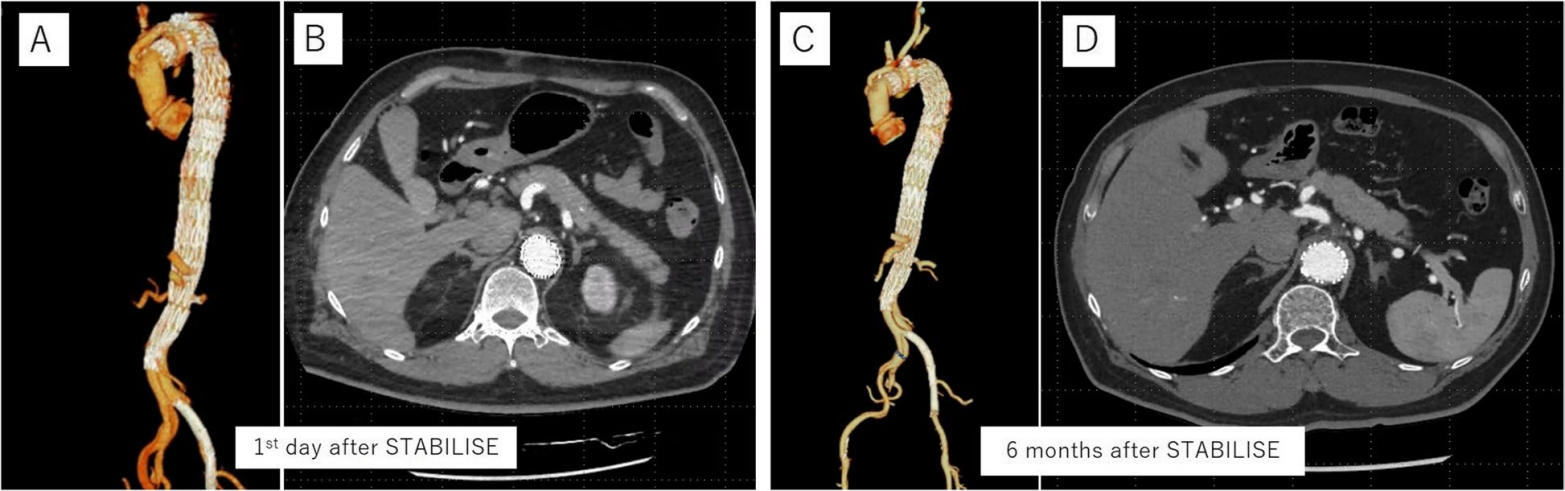
**A**–**D **CT images after STABILISE technique. Legend: **A** and **B**. 3D image (**A**) and axial image (**B**) of the CT scan at the 1st day after STABILISE technique. False lumen of the aorta was obliterated where STABILSE technique was applied. **C** and **D**. 3D image (**C**) and axial image (**D**) of the CT scan at 6 months after STABILISE technique. Good aortic remodeling was maintained without dilation of aorta

Follow-up CT was performed on the 1st day after the STABILISE technique (the 13th day after the initial aortic surgery). The true lumen of the aortic arch to the abdominal aorta was dilated, and the false lumen was completely obliterated where the STABILISE technique was performed (Fig. 3A, B). The patient’s postoperative course was uneventful, and his symptoms resolved. The ABI on the 3rd day after the STABILISE surgery (the 15th day after the initial aortic surgery) was 1.19 on the left side. The patient underwent rehabilitation and was discharged on the 9th day after the STABILISE surgery (the 21st day after the initial aortic surgery). A follow-up CT performed 6 months after the STABILISE technique showed a completely obliterated false lumen and no aortic enlargement (Fig. 3C, D).

## Discussion and conclusions

Acute TAAD with malperfusion occurs in 20.5–33.7% of TAAD cases, of which visceral malperfusion occurs in less than 3%; however, the mortality rate for those with visceral malperfusion is up to 75% [4–6]. Treatment includes primary entry closure, total arch replacement plus open stent grafting, bypass or endovascular therapy for visceral branches, and simultaneous or early thoracic endovascular aortic repair (TEVAR) [7]. Bin et al. reported that total arch replacement plus open stent grafting might improve the prognosis in acute TAAD with malperfusion [8]. However, open stent grafts have been associated with an increased risk of paraplegia due to spinal cord infarctions. An open stent graft may also cause re-dissection due to stent graft-induced new entry (SINE) [9].

In this patient, total arch replacement plus open stent graft (or frozen elephant trunk) was an option. However, since our hospital’s strategy is to prioritize entry resection to keep the treatment simple and minimize surgical stress, only an ascending aorta and partial aortic arch replacement were performed, initially resolving the left limb malperfusion. Unfortunately, the residual false lumen flow resulted in recurrent back pain and intermittent claudication of the left lower limb, requiring additional treatment. The additional treatment options were conventional TEVAR with or without a bare-metal aortic stent or TEVAR with the STABILISE technique. TEVAR is an effective treatment for complicated type B aortic dissection (TBAD), such as rupture and malperfusion. The aim of TEVAR for TBAD is to close the entry tear, which causes a false lumen. TEVAR alone provides good remodeling in the descending thoracic aorta, and TEVAR with a bare-metal aortic stent, known as the PETTICOAT technique, also achieves good thoracic aortic remodeling and good true lumen expansion [10]. However, in both cases, the false lumen at the thoracoabdominal and abdominal aorta remains, as reentry cannot be treated in these segments.

A patent false lumen is associated with complications, such as aortic enlargement and SINE. In addition, impaired blood flow in visceral branches due to a residual false lumen after TEVAR can cause future problems. Furthermore, Faure et al. reported that TEVAR is associated with a 40% re-intervention rate [11]. A residual false lumen after proximal aortic repair is a major problem resulting in a poor prognosis. Therefore, obliteration of the distal aortic false lumen after proximal aortic repair may improve outcomes.

As previously reported, the STABILISE technique can disrupt the intima and obstruct the false lumen, immediately restoring the uniluminal thoracoabdominal aortic flow. In a study by Faure et al. (2018), all patients (*n* = 15) achieved technical success, and the aortic diameter did not expand during the follow-up period (range: 90–735 days). The STABILISE technique has also been used in patients with residual thoracoabdominal dissection after acute TAAD repair, achieving immediate and mid-term persistent remodeling of the thoracoabdominal aorta [12, 13]. In our patient, recurrent symptoms occurred from persistent false lumen flaws; thus, we applied the STABILISE technique, achieving good early results for at least 6 months.

The biggest limitation of the STABILISE technique is that it has not been widely utilized; thus, even though the short- and mid-term results are encouraging, the evidence is limited. For example, in a report by Hofferberth et al., the inclusion criteria were the presence of visceral malperfusion, dissection extending into the aortic branches, proximal false lumen growth (> 5 mm in 12 months), and true lumen collapse [3]. However, in our opinion, these criteria are over-indicated. We believe that most of those cases could have been treated with conventional TEVAR with or without a bare-metal aortic stent, and only persistent false lumen flaws with a high possibility of future complications should be indicated for the STABILISE technique. Therefore, more large-scale studies with long-term follow-ups are required. Furthermore, possibility of aortic injury always remains as we obliterate the intima by dilating a semi-compliant aortic balloon manually, and overdilation can easily occur. We believe development of non-compliant balloon dedicated for STABILISE technique using inflation device is warranted in the future to make this technique feasible for everyone.

In summary, we successfully performed the STABILISE technique on a patient with residual dissection after complications arose from aortic repair for acute TAAD.

## Data Availability

Not applicable.

## Abbreviations

TAAD: Type A aortic dissection
STABILISE: Stent-assisted balloon-induced intimal disruption and relamination of aortic dissection
CT: Computed tomography
EIA: External iliac arteries
BCA: Brachiocephalic artery
CCA: Common carotid artery
SCA: Subclavian artery
CFA: Common femoral artery
ABI: Ankle-brachial pressure index
CIA: Common iliac artery
TEVAR: Thoracic endovascular aortic repair
SINE: Stent-graft-induced new entry
TBAD: Type B aortic dissection
